# When colon cancer crosses boundaries: acute abdomen from direct pancreatic infiltration by a splenic flexure tumour

**DOI:** 10.1093/jscr/rjag715

**Published:** 2026-08-14

**Authors:** Shravani Sripathi, Anna D Coleman, Abhinaya S Krishnaraj, Sundarachalam Pindicura, John P Sharpe

**Affiliations:** Department of Surgery, Central Michigan University, 359 Stoneham Rd, Saginaw, MI 48638, United States; Department of Surgery, Central Michigan University, 359 Stoneham Rd, Saginaw, MI 48638, United States; Department of Surgery, Central Michigan University, 359 Stoneham Rd, Saginaw, MI 48638, United States; Department of Surgery, Central Michigan University, 359 Stoneham Rd, Saginaw, MI 48638, United States; Department of Surgery, Central Michigan University, 359 Stoneham Rd, Saginaw, MI 48638, United States

**Keywords:** colon cancer, splenic abscess, multivisceral resection

## Abstract

Colorectal cancer is the second-leading cause of cancer death in the United States. Locally advanced invasion into an adjacent organ (T4b) occurs in 10%–20% of colon cancers. A 65-year-old man presented with weakness and abdominal pain. The abdomen was soft, moderately distended, and severely tender in the epigastric region. Laboratory studies showed leukocytosis and lactic acidosis. Computed tomography of abdomen demonstrated intra-abdominal free air and splenic abscess. During emergent laparotomy, a perforated splenic flexure mass involving the pancreatic tail and spleen was identified. En bloc resection was performed with temporary abdominal closure. Following hemodynamic improvement, the abdomen was closed with creation of a transverse end colostomy. Pathology revealed moderately differentiated adenocarcinoma with transmural invasion into the pancreatic tail. Multimodal therapy is the standard approach for locally advanced colon cancer. An en-bloc resection of involved organs to achieve an R0 margin remains the cornerstone of surgical management.

## Introduction

Colorectal cancer remains the second-leading cause of cancer-related death in the United States and now ranks first among adults younger than 50 years [1]. A subset of patients present with locally advanced (T4) disease, defined as a tumour penetrating the visceral peritoneum (T4a) or directly invading an adjacent organ or structure (T4b). T4 stage is a major adverse prognostic factor: a large series report 3-year survival of roughly 74%–85% for T4a and 63%–95% for T4b depending on nodal status [2]. Approximately 10%–20% of colon cancers demonstrate adjacent-organ invasion (T4b) at diagnosis, although the rate of true pathologic invasion among clinically suspected T4 tumours is often lower [3].

Tumours arising at the splenic flexure are particularly challenging because of their proximity to the spleen, pancreatic tail, stomach, and abdominal wall [4]. The American Society of Colon and Rectal Surgeons recommends en bloc multivisceral resection with negative margins for locally advanced tumours [5], and National Comprehensive Cancer Network guidelines recommend adjuvant chemotherapy for T4 or node-positive disease [6]. Perforation with splenic abscess formation arising from splenic flexure carcinoma is exceptionally rare, with only a small number of cases reported in the literature [7].

## Case presentation

A 65-year-old man with a significant history of hypertension, type 2 diabetes mellitus, and chronic kidney disease presented to the emergency department after 1 week of fatigue and generalized weakness, along with a 1-day history of worsening abdominal pain. He denied hematochezia, melena, nausea, and vomiting.

On arrival, he was hypotensive and tachycardic. Examination revealed a soft, moderately distended abdomen with severe tenderness in the epigastric region, without overt peritonitis. Laboratory studies were notable for leukocytosis (white blood cell count 17 × 10^9^/L), hemoglobin 10.5 g/dl, lactate 3.4 mmol/L, and creatinine 4.1 mg/dl.

**Figure 1:**
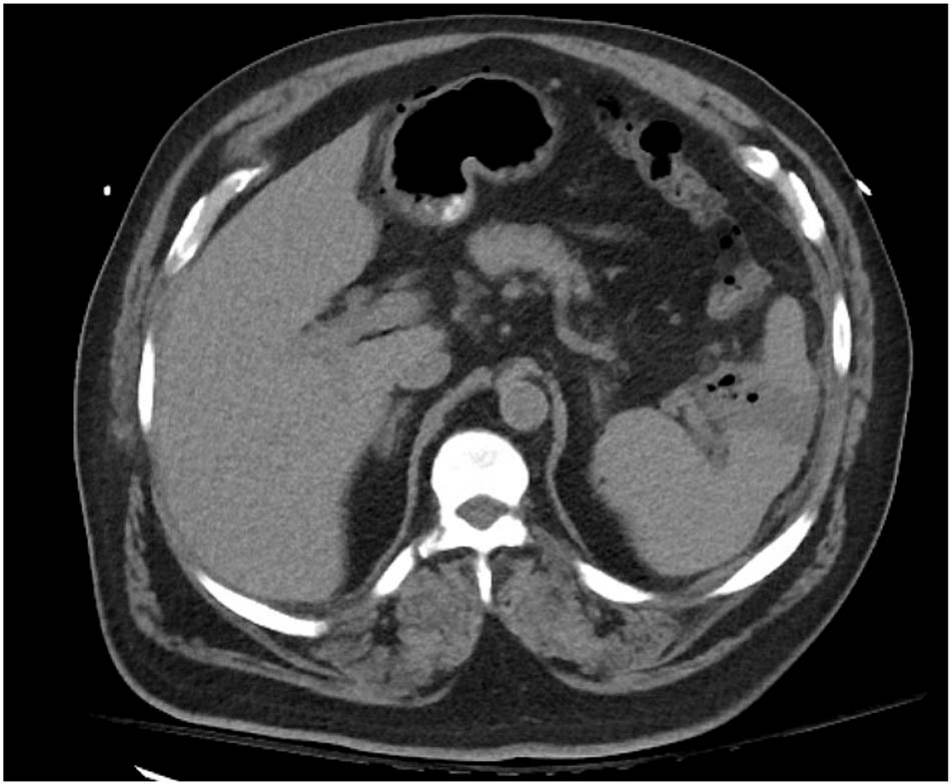
Axial CT section demonstrating the splenic abscess.

**Figure 2:**
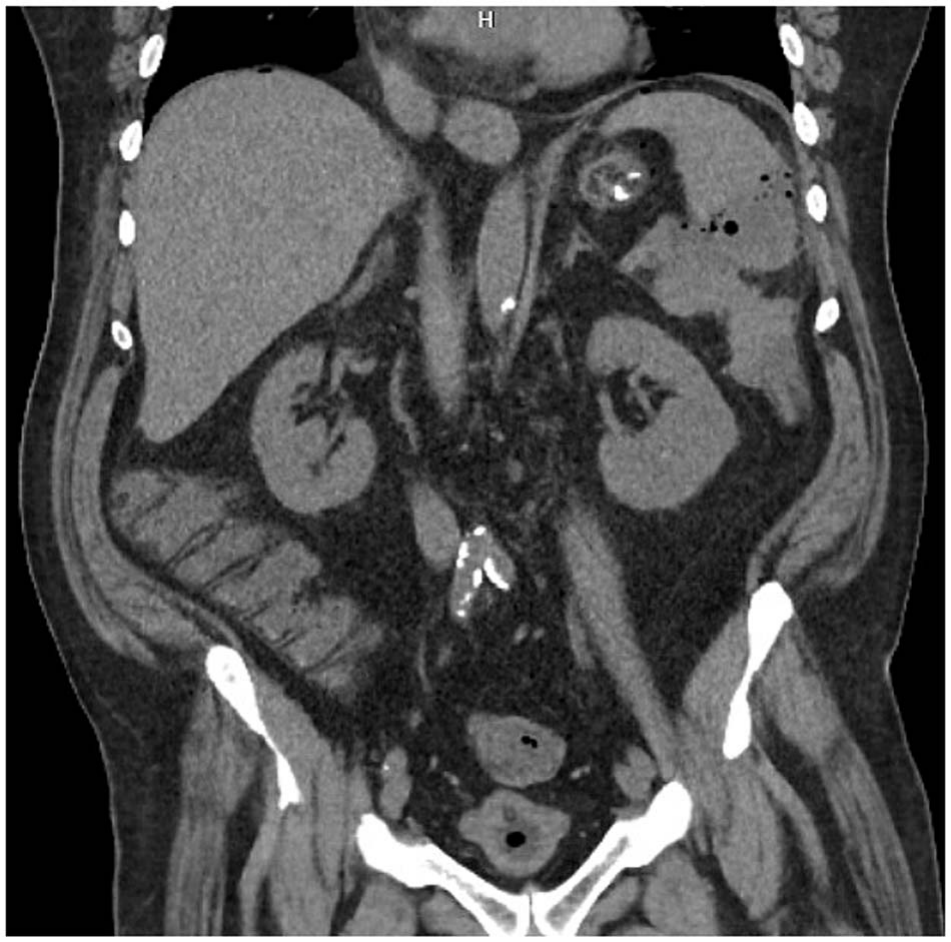
Coronal CT section demonstrating the splenic flexure mass adherent to the pancreatic tail and spleen.

Intravenous fluids and broad-spectrum intravenous antibiotics were initiated because of concern for intra-abdominal sepsis. Non-contrast computed tomography (CT) of the abdomen and pelvis demonstrated intra-abdominal free air and a splenic abscess measuring ~4.5 × 3.2 cm (Figs. 1 and 2). Given the presence of free air and concern for perforation, the patient was taken for emergent exploratory laparotomy. Intraoperatively, a perforated mass at the splenic flexure was identified with apparent involvement of the pancreatic tail; no other abnormalities were noted. An en bloc resection of the left colon, distal pancreas, and spleen was performed. Because of continued hemodynamic instability and vasopressor requirements, a damage-control strategy was adopted: the abdomen was temporarily closed, and the patient was transferred to the intensive care unit.

On postoperative day 1, vasopressor requirements decreased, and the patient returned to the operating room for second-look laparotomy. The remaining bowel appeared healthy, a transverse end colostomy was created, the midline laparotomy was closed, and a Jackson-Pratt (JP) drain was placed. He was extubated on postoperative day 2. Renal function improved gradually, diet was advanced as tolerated, and the colostomy functioned appropriately. Final pathology revealed moderately differentiated adenocarcinoma of the splenic flexure with transmural invasion extending into the pancreatic tail; 1 of 20 examined lymph nodes was positive for metastatic carcinoma, corresponding to a pathologic stage of pT4bN1a. The carcinoembryonic antigen level was <5 ng/ml. Oncology recommended adjuvant chemotherapy. The patient received the recommended post-splenectomy vaccinations, the JP drain was removed once output decreased appropriately, and he was discharged home in stable condition with outpatient surgical and oncologic follow-up.

## Discussion

T4b colon cancers represent a challenging subset of colorectal malignancies because of frequent involvement of adjacent organs. The single most important determinant of long-term survival is achieving an R0 (margin-negative) resection [8], which frequently requires complex en bloc multivisceral resection. Contemporary multicenter data indicate that multivisceral resection for T4b colon cancer carries postoperative complication rates exceeding 30%, with 5-year overall survival of ~50%, and that nodal status rather than the specific organ resected is the dominant prognostic factor [9].

In this patient, septic shock from a perforated splenic flexure tumour with associated splenic abscess produced profound hemodynamic instability, necessitating temporary abdominal closure followed by staged re-exploration. This damage-control approach is well established in critically ill non-trauma surgical patients and can reduce postoperative complications in the setting of severe sepsis by prioritizing source control and physiologic restoration before definitive reconstruction [10]. Perforated colon cancer carries a recognized double jeopardy of septic insult superimposed on malignant disease, with perioperative mortality reported ~12% and worse outcomes for free compared with contained perforation; current evidence supports prioritizing septic source control acutely while still pursuing an oncologically oriented resection when feasible [11].

T4b colon cancer perforating with splenic abscess formation is an extremely rare clinical entity, with only a few documented cases reported in the literature [7]. The proposed mechanism involves direct tumour invasion through the colonic wall into the spleen, formation of a splenocolic fistula at the site of tumour ulceration, migration of enteric flora through the fistula, and subsequent splenic abscess [7], [12].

Pancreatic invasion in T4b colon cancer is likewise uncommon, occurring in roughly 5%–15% of multivisceral resections for locally advanced colon cancer, and the pancreas is among the less frequently involved organs relative to the abdominal wall, small bowel, and bladder [13, 14]. When pancreatic involvement is present, the surgical approach depends on tumour location along the gland. En bloc pancreatic resection for T4b colon cancer carries substantial morbidity—one contemporary series reported major complications (Clavien–Dindo grade III–V) in 34.4% of pancreas-involving multivisceral resections—yet long-term survival remains favourable, with 5-year survival of ~55%–66% and nodal status again the strongest predictor of outcome [15].

## Conclusion

This case represents a rare presentation of perforated splenic flexure adenocarcinoma invading the pancreatic tail and spleen, complicated by splenic abscess formation and septic shock. Emergent surgical intervention was required to stabilize the patient and achieve infectious source control. En bloc multivisceral resection remains the cornerstone of surgical management for T4b colon cancer with adjacent-organ invasion; although associated with significant morbidity, it offers the best opportunity for long-term survival when an R0 margin is achieved. Early multidisciplinary involvement is essential in the management of complicated, locally advanced colon cancer.
